# AI in Medical Questionnaires: Scoping Review

**DOI:** 10.2196/72398

**Published:** 2025-06-23

**Authors:** Xuexing Luo, Yiyuan Li, Jing Xu, Zhong Zheng, Fangtian Ying, Guanghui Huang

**Affiliations:** 1 Faculty of Humanities and Arts Macau University of Science and Technology Macau China; 2 Industrial and Manufacturing Engineering European Academy of Engineering Gothenburg Sweden; 3 College of Computer Science and Technology Zhejiang University Hangzhou China; 4 Zhuhai M.U.S.T. Science and Technology Research Institute Zhuhai China

**Keywords:** artificial intelligence, AI, medical questionnaires, questionnaire-based prediction, questionnaire development, diagnostic accuracy

## Abstract

**Background:**

The World Health Organization reports that >1 billion people worldwide experience mental disorders, with the prevalence of depression and anxiety among children and adolescents at 2.6% and 6.5%, respectively. However, commonly used clinical questionnaires such as the Hamilton Depression Rating Scale and the Beck Depression Inventory suffer from several problems, including the high degree of overlap of symptoms of depression with those of other psychiatric disorders and a lack of professional supervision during administration of the questionnaires, which often lead to inaccurate diagnoses. In the wake of the COVID-19 pandemic, the health care system is facing the dual challenges of a surge in patient numbers and the complexity of mental health issues. Artificial Intelligence (AI) technology has now been shown to have great promise in improving diagnostic accuracy, assisting clinical decision-making, and simplifying questionnaire development and data analysis.

**Objective:**

This review aimed to explore the current applications, potential benefits, and issues of AI in medical questionnaires, focusing on its role in 3 main functions: assessment, development, and prediction. The global mental health burden remains severe.

**Methods:**

The review included peer-reviewed studies that applied AI technologies to medical, psychological, or physiological questionnaires and reported measurable outcomes; non–peer-reviewed, non-English/Chinese, ethically noncompliant, or AI-unrelated studies were excluded. Five databases (PubMed, Embase, Cochrane Library, Web of Science, and CNKI) were searched from inception through September 2024. Three independent reviewers conducted data extraction, quality appraisal using the Joanna Briggs Institute tools, and narrative synthesis of AI applications across questionnaire assessment, development, and prediction tasks.

**Results:**

Of 49,091 publications, a total of 14 (0.03%) studies met the inclusion criteria. AI technologies showed advantages in assessment, such as distinguishing myalgic encephalomyelitis or chronic fatigue syndrome from long COVID-19 with 92.18% accuracy. In questionnaire development, natural language processing using generative models such as ChatGPT was used to construct culturally competent scales. In terms of disease prediction, one study had an area under the curve of 0.790 for cataract surgery risk prediction. Overall, 24 AI technologies were identified, covering traditional algorithms such as random forest, support vector machine, and k-nearest neighbor, as well as deep learning models such as convolutional neural networks, Bidirectional Encoder Representations From Transformers, and ChatGPT. Despite the positive findings, only 21% (3/14) of the studies had entered the clinical validation phase, whereas the remaining 79% (11/14) were still in the exploratory phase of research. Most of the studies (10/14, 71%) were rated as being of moderate methodological quality, with major limitations including lack of a control group, incomplete follow-up data, and inadequate validation systems.

**Conclusions:**

In summary, the integrated application of AI in medical questionnaires has significant potential to improve diagnostic efficiency, accelerate scale development, and promote early intervention. Future research should pay more attention to model interpretability, system compatibility, validation standardization, and ethical governance to effectively address key challenges such as data privacy, clinical integration, and transparency.

## Introduction

According to World Health Organization (WHO) data from 2022, approximately 1 billion individuals worldwide experience mental disorders [1,2]. Worldwide, the prevalence of depression and anxiety disorders among children and adolescents is estimated at approximately 2.6% and 6.5%, respectively [3,4]. This high disease burden necessitates reliable screening tools, yet current gold-standard questionnaires face two critical challenges. First, although these authoritative psychological assessment tools are used during screening and diagnosis, depressive symptoms often overlap with those of other psychiatric disorders, such as bipolar affective disorder and obsessive-compulsive disorder [5-9], making accurate diagnosis difficult when relying on a single questionnaire. Second, in many health care settings, patients complete questionnaires without proper guidance or oversight due to inadequate clinical supervision, resulting in distorted outcomes when various psychological and physiological self-assessment tools are applied in practice [10-12]. These limitations continually underscore the inefficiencies in the diagnostic utility and administration of traditional medical questionnaires. To obtain accurate health data and assist physicians during patient consultations, there is an urgent need to explore more efficient and precise assessment techniques. This study aimed to investigate how artificial intelligence (AI) technologies can address these fundamental limitations of traditional assessment tools while enhancing clinical decision-making processes.

Following the outbreak of COVID-19 in 2019, hospitals have faced a dual challenge of surging patient volumes and increasingly complex mental health care needs [13-15]. These unprecedented circumstances have exposed the inadequacies of traditional assessment approaches, creating an opportunity for technological innovation in mental health care delivery. Advancements in machine learning (ML) and large language models (LLMs) have demonstrated significant potential to reduce analytical biases, support clinical decision-making, and improve data processing efficiency [16-20], drawing medical experts’ attention to how intelligent technologies can compensate for the inefficiency and subjectivity of traditional scales. Since 2013, the integration of ML with LLMs has resulted in breakthroughs in natural language processing (NLP) [21], complex reasoning, multilingual support, and multidimensional data analysis [22-26]. By 2024, these developments evolved into specialized mental health–oriented LLMs capable of identifying stress, depression, and suicidal ideation, thereby facilitating disease screening and early intervention [27-31]. This technological evolution directly responds to the postpandemic challenges by offering more accurate, efficient, and accessible screening tools that can identify psychological conditions early and create critical windows for timely intervention [32-34].

Multiple research studies across different application domains have substantiated the technological advantages of AI in mental health assessment. On the basis of the developments described previously, AI systems demonstrate several key capabilities that directly address the limitations of traditional questionnaires. First, ML algorithms excel at detecting subtle patterns across multiple variables that human clinicians might miss, as demonstrated by the high accuracy of the study by McGarrigle et al [35] in distinguishing among myalgic encephalomyelitis or chronic fatigue syndrome, post–COVID-19 condition, and healthy controls using random forest (RF) algorithms. LLMs’ NLP capabilities capture contextual meanings and emotional undertones in patient narratives that structured questionnaires cannot. Unlike traditional categorical approaches, ML enables dimensional symptom representation, aligning with contemporary understanding of spectrum disorders, as shown in the approach by De Luca et al [36] to modeling suicide risk. In addition, AI-based systems implement adaptive questioning pathways that enhance user satisfaction while maintaining diagnostic validity, as Nam et al [37] demonstrated with their conversational AI for spinal pain assessment. Finally, AI can integrate multimodal data, as evidenced by the improvement in pathological voice assessment by Kojima et al [38] by combining acoustic features with questionnaire responses. These capabilities directly address traditional questionnaires’ limitations in handling symptom overlap and contextual interpretation, providing a foundation for the practical applications of AI in clinical settings that will be discussed in the following section.

The comprehensive technical advantages are obvious. Rapid developments in ML, data science, and neural networks have allowed AI to be involved in the evaluation, development, and predictive modeling phases of medical questionnaires within clinical practice [39-41]. With its efficiency, accuracy, and capacity to handle large-scale data, AI enables clinicians and health care professionals to swiftly access and evaluate patient data derived from medical questionnaires, thereby improving primary health care efficiency [42-45]. For instance, advances in NLP have supported rapid screening for depression and anxiety, reducing initial consultation times [21,46-48], whereas deep learning (DL) has contributed to a range of medical tasks, including large-scale health data screening [49-59], pathological segmentation [53-56], and disease monitoring [57-59]. Integrating LLMs with traditional psychological assessments can reduce human error and ensure consistency in professional diagnoses. By 2024, core NLP technologies integrated with ML methods were being used to classify depression and its severity [60].

This paper identifies key limitations in traditional medical scales, including substantial diagnostic bias, low screening efficiency, and data distortion. It explores the potential applications of AI in the health care domain, focusing on how AI-driven approaches can enhance evaluation, development, and prediction—3 critical diagnostic stages—while examining recent algorithmic research and clinical findings. In addition, it summarizes the impacts of AI-augmented traditional questionnaires on patient outcomes. Finally, it addresses broader societal and ethical considerations, such as privacy, fairness, transparency, and ethical challenges. This review concludes by discussing future developmental trajectories and scientific hypotheses concerning the integration of AI into medical questionnaires.

## Methods

### Data Sources and Search Strategies

This study was designed according to the latest version of the PRISMA-ScR (Preferred Reporting Items for Reviews and Meta-Analyses extension for Scoping Review) checklist [61] (Multimedia Appendix 1). This study was conducted through a systematic literature search in several databases (PubMed, Embase, Cochrane Library, Web of Science, and China National Knowledge Infrastructure on the China Knowledge Network) with the aim of exploring the innovation, diagnosis, and impact of AI in medical scales. The search keywords and other related terms in this study are shown in Table 1. In addition, the search was conducted from the inception of each database to September 2024 to ensure the inclusion of the latest relevant studies and was limited to English- and Chinese-language literature.

**Table 1:** Search strategies for English- and Chinese-language databases.

	Search term
1	“Artificial intelligence” (MeSH^a^)
2	“Machine learning” (MeSH)
3	“Deep learning” (MeSH)
4	1 OR 2 OR 3
5	“Questionnaire”
6	“Scale”
7	“Medical questionnaire”
8	“Medical scale”
9	“Psychological questionnaire”
10	“Psychological scale”
11	“Physiological questionnaire”
12	“Physiological scale”
13	“Mental questionnaire”
14	“Mental scale”
15	5 OR 6 OR 7 OR 8 OR 9 OR 10 OR 11 OR 12 OR 13 OR 14
16	4 AND 15
17	“Rengongzhineng” (artificial intelligence)
18	“Jiqixuexi” (machine learning)
19	“Shenduxuexi” (deep learning)
20	17 OR 18 OR 19
21	“Diaochawenjuan” (questionnaire)
22	“Liangbiao” (scale)
23	“Yixue diaochawenjuan” (medical questionnaire)
24	“Yixue liangbiao” (medical scale)
25	“Xinli diaochawenjuan” (physiological questionnaire)
26	“Xinli liangbiao” (psychological scale)
27	“Shengli diaochawenjuan” (physiological questionnaire)
28	“Shengli liangbiao” (physiological scale)
29	“Jingshen diaochawenjuan” (mental questionnaire)
30	“Jingshen liangbiao” (mental scale)
31	21 OR 22 OR 23 OR 24 OR 25 OR 26 OR 27 OR 28 OR 29 OR 30
36	20 AND 31

^a^MeSH: Medical Subject Headings.

### Inclusion and Exclusion Criteria

The inclusion criteria for the studies can be specifically divided into three categories: (1) studies related to the application of AI technology to disease management, psychological, or physiological questionnaires; (2) articles that provided relevant data to support and validate the effectiveness of the application of AI technology to questionnaires; and (3) peer-reviewed literature, which may include but was not limited to randomized controlled trials, cohort studies, reviews, meta-analyses, and cross-sectional studies. On the basis of the inclusion criteria, the exclusion criteria for this review were as follows: (1) studies that did not use AI technologies and did not involve medical questionnaires; (2) literature in the category of *gray literature*, such as non–peer-reviewed literature, unpublished manuscripts, or conference abstracts; (3) literature not in English or Chinese (the authors’ native language) and literature in Chinese that did not meet the inclusion criteria; (4) experimental literature that did not have ethics approval or obtain informed consent; (5) articles on the uses of AI not being ethical; and (6) articles on conflicts of interest related to AI technology.

### Data Charting and Synthesis

In this study, a comprehensive literature screening and evaluation process was implemented to ensure the objectivity and accuracy of the research. This process was executed by 3 independent reviewers: YL, JX, and XL. Initially, YL was responsible for downloading and conducting a preliminary review of the screened literature to exclude documents unrelated to the study’s topic. Subsequently, the relevant literature from the preliminary screening was passed to JX for a more detailed eligibility assessment. The included literature was then double-checked by YL and JX. In cases of disagreement between YL and JX, XL made the final decision. Only literature agreed upon by all 3 reviewers was included in the final analysis. Furthermore, throughout the data extraction process, the 3 reviewers (XL, YL, and JX) created 5 distinct tables and figures, each with a specific function, to systematically organize and analyze the collected data. The primary purpose of creating these tables and figures was to enhance the transparency, systematic approach, and scientific rigor of this review by organizing and analyzing data in a standardized manner. Table 1 presents the search strategies and keywords used in both English- and Chinese-language databases.

### Quality Evaluation Methods

To ensure the quality of the selected literature, this review used the Joanna Briggs institute (JBI) critical appraisal tools [62]. The final quality assessment of the included literature was conducted using the scoring system provided by the JBI guidelines. This system assigns 1 point for each criterion fully met, with a score of 1 for *yes* and 0 for *no* or *unclear* responses. This scoring method facilitates a horizontal comparison of study quality, allowing for ranking based on the total score. For qualitative research, studies with <5 points were considered low quality, studies with 5 to 7 points were considered moderate quality, and studies with ≥8 points were considered high quality. For review studies, scores of <6 points indicated low quality, scores of 6 to 8 points indicated moderate quality, and scores of ≥9 points indicated high quality. Given the specific design and implementation of quasi-experimental studies, the scoring criteria were adjusted accordingly—scores of <5 points indicated low quality, scores of 5 to 7 points indicated moderate quality, and scores of ≥8 points indicated high quality. This systematic assessment approach ensured the reliability and scientific rigor of the review findings.

### Quality Assessment

#### JBI Checklist for Systematic Reviews and Research Syntheses

The items in this checklist were as follows: (1) is the review question clearly and explicitly stated? (2) Were the inclusion criteria appropriate for the review question? (3) Was the search strategy appropriate? (4) Were the sources and resources used to search for studies adequate? (5) Were the criteria for appraising the studies appropriate? (6) Was critical appraisal conducted by ≥2 reviewers independently? (7) Were there methods to minimize errors in data extraction? (8) Were the methods used to combine studies appropriate? (9) Was the likelihood of publication bias assessed? (10) Were recommendations for policy or practice supported by the reported data? (11) Were the specific directives for new research appropriate?

#### JBI Checklist for Quasi-Experimental Studies

The items in this checklist were as follows: (1) is it clear in the study what is the *cause* and what is the *effect* (ie, there is no confusion about which variable comes first)? (2) Was there a control group? (3) Were participants included in any comparisons similar? (4) Were the participants included in any comparisons receiving similar treatment or care other than the exposure or intervention of interest? (5) Were there multiple measurements of the outcome both before and after the intervention or exposure? (6) Were the outcomes of participants included in any comparisons measured in the same way? (7) Were outcomes measured in a reliable way? (8) Was follow-up complete and, if not, were differences between groups in terms of their follow-up adequately described and analyzed? (9) Was appropriate statistical analysis used?

#### JBI Checklist for Qualitative Research

The items in this checklist were as follows: (1) is there congruity between the stated philosophical perspective and the research methodology? (2) Is there congruity between the research methodology and the research question or objectives? (3) Is there congruity between the research methodology and the methods used to collect data? (4) Is there congruity between the research methodology and the representation and analysis of the data? (5) Is there congruity between the research methodology and the interpretation of the results? (6) Is there a statement locating the researcher culturally or theoretically? (7) Is the influence of the researcher on the research and vice versa addressed? (8) Are participants, and their voices, adequately represented? (9) Is the research ethical according to current criteria or, for recent studies, is there evidence of ethics approval by an appropriate body? (10) Do the conclusions drawn in the research report flow from the analysis or interpretation of the data?

## Results

### Overview

**Figure 1:**
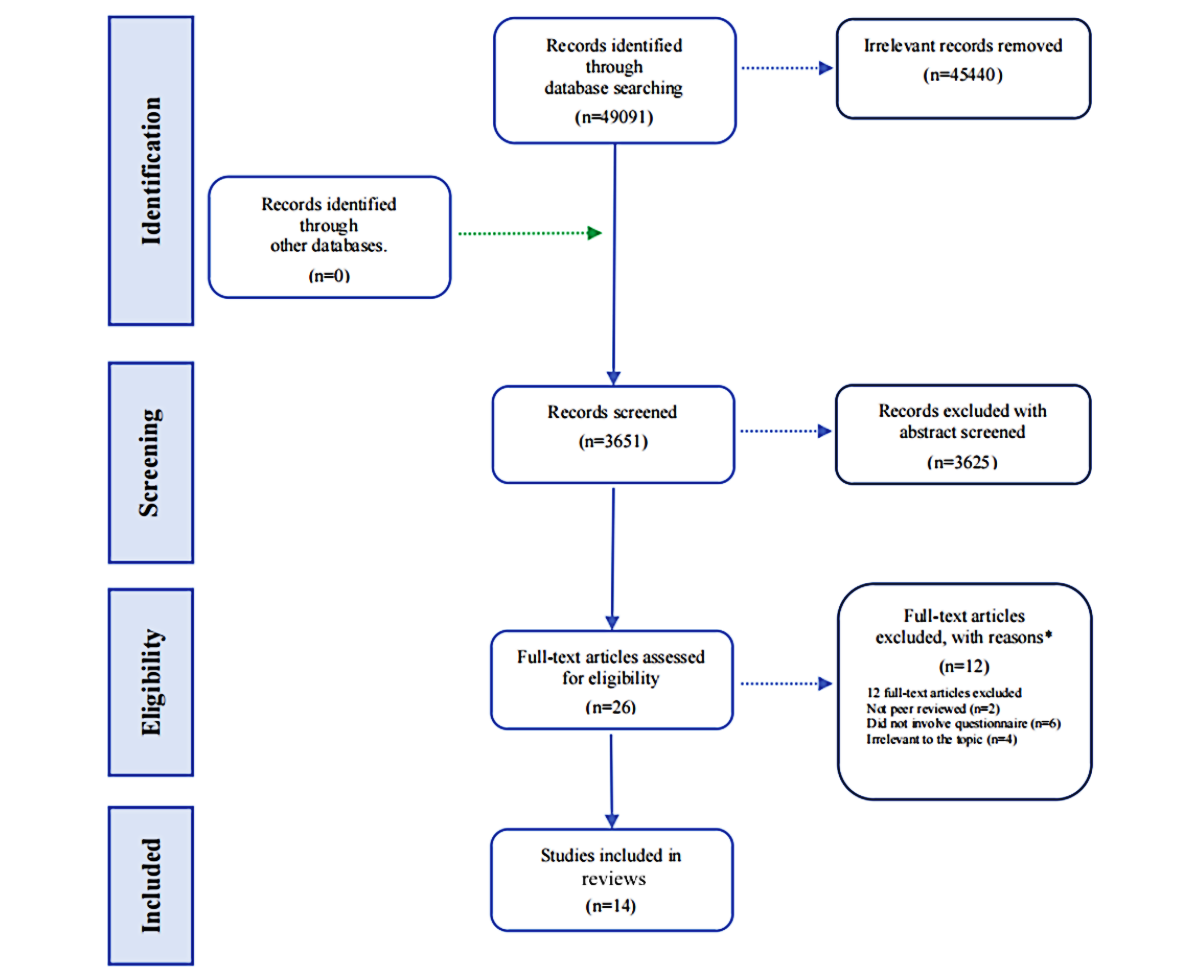
Flow diagram for the included and excluded articles.

The screening process for inclusion of studies is shown in Figure 1. An initial 49,091 records were identified through a systematic database search. After removing duplicates and excluding irrelevant articles, of the 49,091 initial articles, 3651 (7.44%) remained for title and abstract screening. After this step, of the 3651 articles, 3625 (99.29%) were excluded. The remaining 0.71% (26/3651) of the articles were assessed in full text and included various types of reviews, clinical studies, and case reports. Of these 26 articles, 12 (46%) were excluded because they did not meet the inclusion criteria. These reasons included a lack of peer review, failure to use questionnaire methodology, and insufficient relevance to the focus of the study. Ultimately, 14 articles were included in the final review.

**Figure 2:**
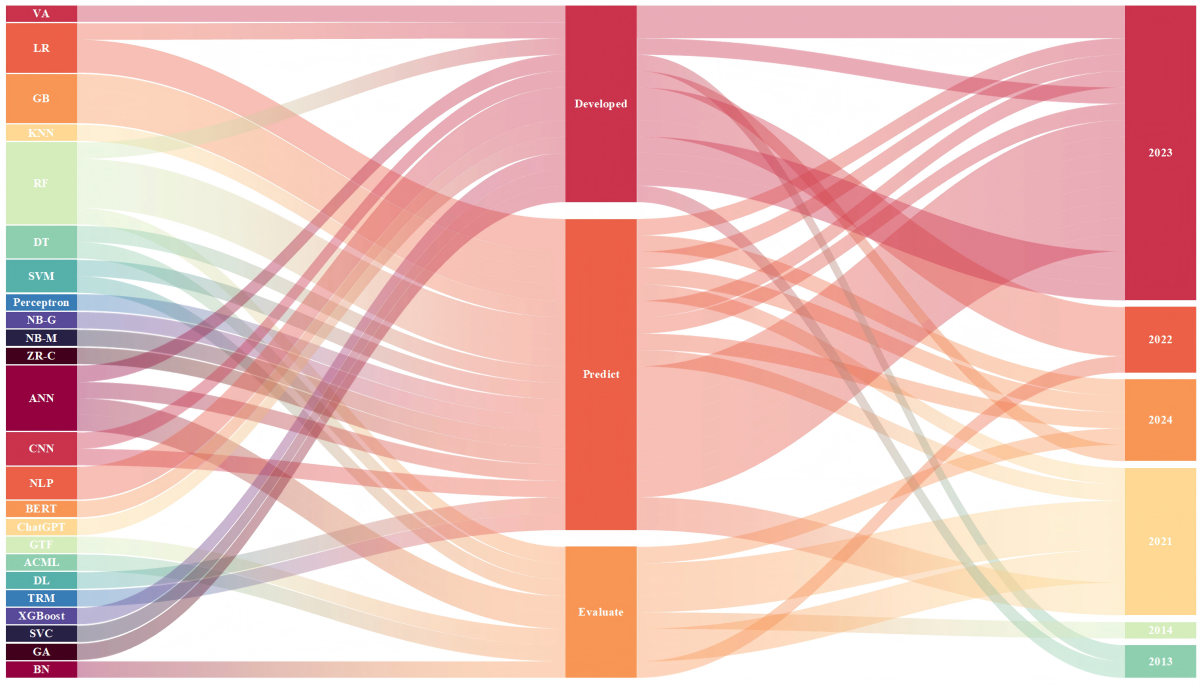
2013-2023 distribution of 24 different artificial intelligence technologies (machine learning and deep learning) in the diagnosis of physiological and psychological conditions based on medical questionnaires. ACML:Apple’s Create ML; ANN: artificial neural network; BERT: bidirectional encoder representations from transformers; BN: Bayesian network; CNN: convolutional neural network; DL: multilayer feedforward deep learning; DT: decision tree; GA: genetic algorithm; GB: gradient boosting; GTF: Google’s TensorFlow; KNN: k-nearest neighbor; LR: logistic regression; NB-G: naive Bayes–Gaussian; NB-M: naive Bayes–multinomial; NLP: natural language processing; RF: random forest; SVC: support vector classifier; SVM: support vector machine; TRM: traditional regression model; VA: voting algorithm; XGBoost: extreme gradient boosting; ZR-C: ZeroR classifier.

**Figure 3:**
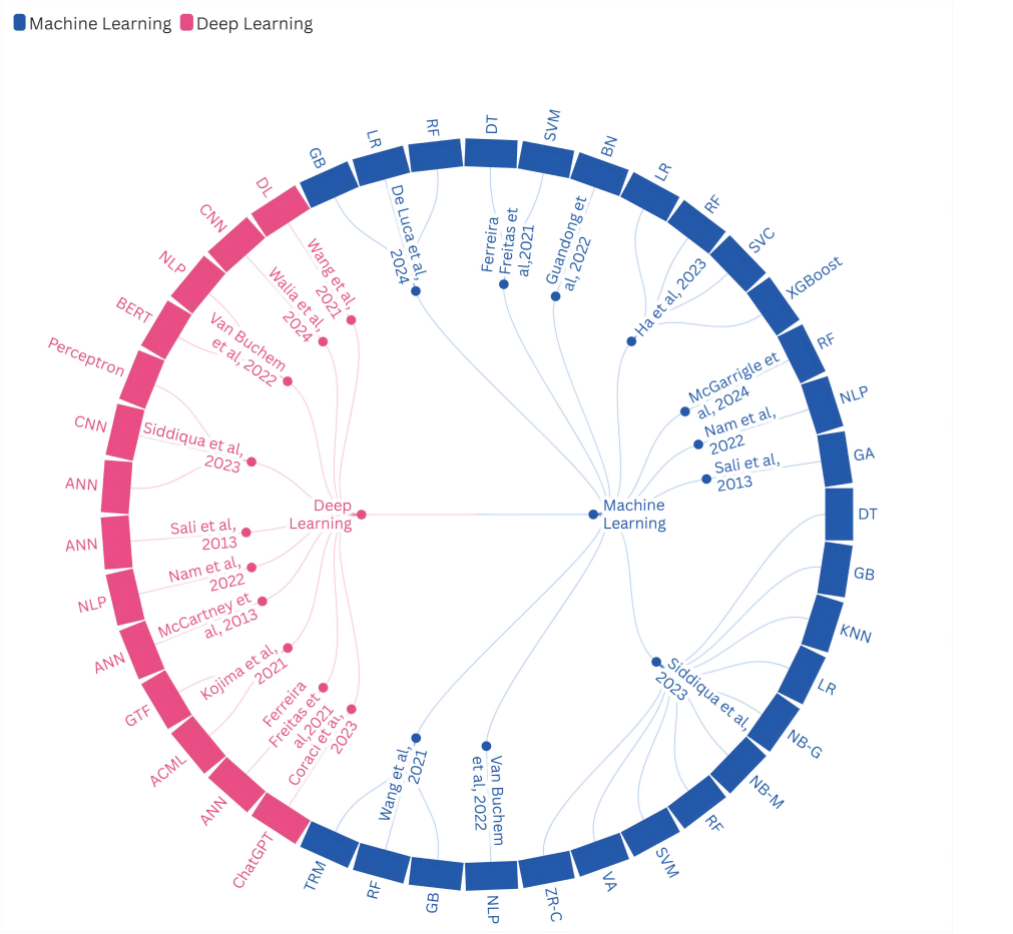
Distribution of machine learning and deep learning technologies across the studies. ACML: Apple’s Create ML; ANN: artificial neural network; BERT: bidirectional encoder representations from transformers; BN: Bayesian network; CNN: convolutional neural network; DL: multilayer feedforward deep learning; DT: decision tree; GA: genetic algorithm; GB: gradient boosting; GTF: Google’s TensorFlow; KNN: k-nearest neighbor; LR: logistic regression; NB-G: naive Bayes–Gaussian; NB-M: naive Bayes–multinomial; NLP: natural language processing; RF: random forest; SVC: support vector classifier; SVM: support vector machine; TRM: traditional regression model; VA: voting algorithm; XGBoost: extreme gradient boosting; ZR-C: ZeroR classifier.

Table 2 assesses the quality of the studies using standardized JBI tools, categorizing the studies into low, medium, and high quality. Figure 2 shows the distribution of 24 different AI technologies (ML and DL) in the diagnosis of physiological and psychological conditions based on questionnaires. Multimedia Appendix 2 details the application of intelligent technologies in clinical and research environments (N=14), emphasizing how different AI methods facilitate the development, prediction, and evaluation of questionnaires. Finally, Figure 3 illustrates the distribution patterns of ML and DL technologies across the various studies. This framework ensured comprehensive data extraction, quality control, and systematic synthesis of AI applications in the implementation of medical questionnaires.

**Table 2:** Summary of the quality evidence in the included 14 reports.

Study	Study design	Assessment of the quality of the study														Overall score
		1	2	3	4	5	6	7	8	9	10	11	12	13		
Siddiqua et al [63], 2023	Quasi-experimental study	Yes	No	Yes	Unclear	No	Yes	Yes	No	Yes	—^a^	—	—	—	Medium quality (5/9)	
van Buchem et al [64], 2022	Qualitative research	Yes	Yes	Yes	Yes	Yes	No	No	Yes	Yes	Yes	—	—	—	Medium quality (8/10)	
Coraci et al [65], 2023	Quasi-experimental study	Yes	Yes	Yes	Yes	No	Yes	Yes	Unclear	Yes	—	—	—	—	Medium quality (7/9)	
Nam et al [37], 2022	Quasi-experimental study	Yes	No	Yes	Yes	No	Yes	Yes	Unclear	Yes	—	—	—	—	High quality (6/9)	
De Luca et al [36], 2024	Systematic review and research synthesis	No	No	No	No	No	No	Yes	No	No	Yes	Yes	—	—	Low quality (3/11)	
McGarrigle et al [35], 2024	Quasi-experimental study	Yes	Yes	Unclear	Yes	No	Yes	Yes	No	Yes	—	—	—	—	Medium quality (6/9)	
Kojima et al [38], 2024	Quasi-experimental study	Yes	No	Yes	Yes	No	Yes	Yes	No	Yes	—	—	—	—	Medium quality (6/9)	
Wang et al [66], 2021	Systematic review and research synthesis	No	No	No	No	No	No	Yes	No	No	Yes	Yes	—	—	Low quality (3/11)	
Ha et al [67], 2023	Quasi-experimental study	Yes	Yes	Unclear	Yes	No	Yes	Yes	No	Yes	—	—	—	—	Medium quality (6/9)	
Shetty et al [68], 2024	Quasi-experimental study	Yes	No	Yes	Yes	No	Yes	Yes	No	Yes	—	—	—	—	Medium quality (6/9)	
McCartney et al [69], 2014	Quasi-experimental study	Yes	No	Unclear	Yes	No	Yes	Yes	No	Yes	—	—	—	—	Medium quality (5/9)	
Sali et al [70], 2013	Quasi-experimental study	Yes	No	Yes	Yes	No	Yes	Yes	No	Yes	—	—	—	—	Medium quality (6/9)	
Ferreira Freitas et al [71], 2021	Quasi-experimental study	Yes	No	Yes	Yes	No	Yes	Yes	No	Yes	—	—	—	—	Medium quality (6/9)	
Li et al [72], 2022	Quasi-experimental study	Yes	No	Unclear	No	No	Yes	Yes	No	Yes	—	—	—	—	Low quality (4/9)	

^a^Not applicable.

### Quality Assessment

The quality of the 14 included studies was assessed using the JBI critical appraisal tools (Table 2). Each study was evaluated using the appropriate checklist based on its design (Table 2)—the JBI critical appraisal checklist for experimental studies (11 items), the JBI qualitative research checklist (1 item), or the JBI systematic review and research synthesis checklist (2 items). Most of the included studies (10/14, 71%) were of moderate methodological quality, with only 7% (1/14) of the studies rated as high quality [66] and 21% (3/14) of the studies classified as low quality [36,66,72]. Methodological strengths commonly observed in quasi-experimental studies included clear causal relationships, reliable outcome measurements, and appropriate statistical analyses. However, the absence of control groups and incomplete descriptions of follow-up were common limitations. A total of 14% (2/14) of the studies, which were systematic reviews, identified flaws in the search strategies and the critical appraisal methodology. This quality assessment provided essential context for interpreting the findings related to the application of AI in medical questionnaires and highlighted the need for more rigorous methodological validation in this rapidly evolving field. Overall, the predominance of moderate-quality studies indicates significant room for improvement in study design and reporting.

### AI and Medical Questionnaires

#### Overview

**Figure 4:**
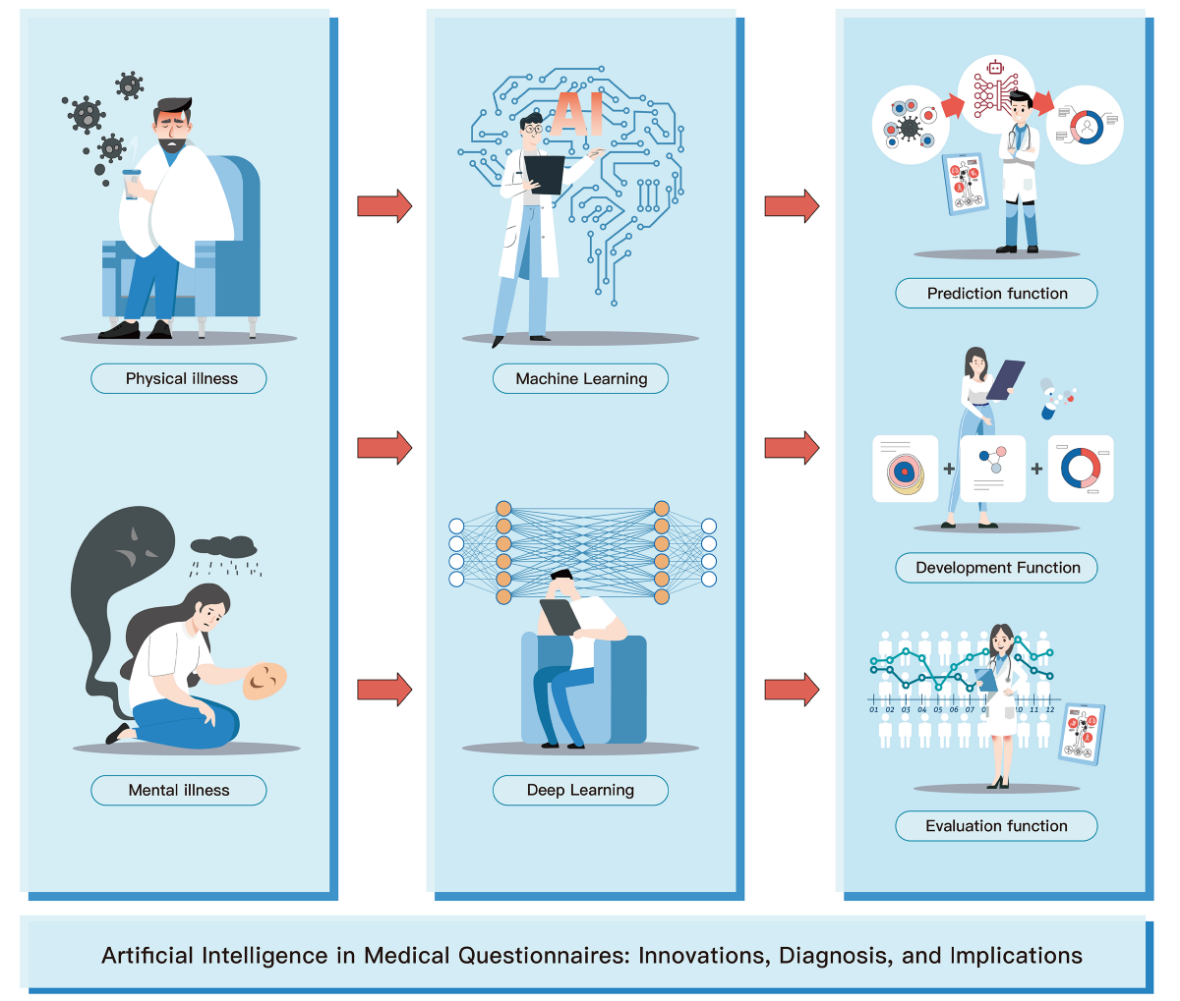
Artificial intelligence in medical questionnaires.

The use of AI for medical questionnaires harnesses DL and ML to enhance disease evaluation, facilitate the development of novel questionnaires, and improve data predictive capacities. These 3 facets are essential diagnostic stages for assessing both physiological conditions and psychological issues. By reviewing recent algorithmic advances and clinical practice, this study uncovered the potential of incorporating AI into traditional medical questionnaires, offering new perspectives for broader clinical applications and informed medical diagnoses (Figure 4).

#### Enhancing the Efficiency of Medical Questionnaire Assessments Through AI

According to WHO data, since the onset of the COVID-19 pandemic, the global population experiencing anxiety and depression has markedly increased, accompanied by a 40% surge in the use of standardized questionnaires [73-76]. Traditional questionnaires offer convenient dissemination and, in some cases, self-administration [77-79]. However, as definitive diagnoses still require physician consultations, the assessment process remains labor intensive. Due to its speed and accuracy in data processing, AI has progressively been integrated into clinical support [43,44]. Notably, DL and NLP technologies—such as the pretrained bidirectional encoder representations from transformers (BERT) model and conversation-based models such as ChatGPT—observed rising adoption rates from 2023 to 2024 [77-80]. Furthermore, traditional algorithms, including support vector machine and *k*-nearest neighbor, continue to effectively evaluate patient data for complex disorders. Recent developments in the technical frameworks for applying these methods to medical questionnaire assessments are illustrated in Figure 2.

Analysis of AI applications in medical questionnaires showed that only 21% (3/14) of the studies involved AI-assisted questionnaires that had entered the clinical validation stage, whereas the remaining 79% (11/14) of the articles described the technology as being in the research stage (Multimedia Appendix 2). Clinical validation was been achieved in patient experience assessment using NLP-based sentiment analysis, in spinal pain evaluation through conversational AI systems [37], and in surgical risk prediction using ML for cataract surgery [66]. These applications share the characteristics of established clinical workflows, model interpretability, and structured validation frameworks. Most AI applications in mental health assessment, pain evaluation, disease differentiation, and specialized assessments remain in the research phases despite promising performance. This pattern stems from psychological assessment complexity [81], data standardization challenges, stringent clinical implementation requirements [82], and emerging technologies’ nascent nature in medical contexts. The findings indicate that AI-assisted medical questionnaires are technically feasible but still transitioning from research innovation to clinical implementation.

#### Enhancing the Efficiency of Dynamic Assessments

By leveraging DL on patient data, AI effectively evaluates how medical questionnaires adaptively distinguish between patient symptom variations, thereby accurately capturing changes in health status and demonstrating advantages in dynamic assessment. During prediagnostic evaluations, many pathological conditions have overlapping manifestations. For instance, post–COVID-19 sequelae resemble acute COVID-19 symptoms, and depression in bipolar disorders overlaps with that observed in unipolar depression [83-86]. To enhance diagnostic accuracy, one study used a series of RF algorithms to assess the psychometric properties of the DePaul Symptom Questionnaire–Short Form in classifying individuals with post–COVID-19 sequelae, those with myalgic encephalomyelitis or chronic fatigue syndrome (unrelated to COVID-19), and healthy controls. The results indicated that the DePaul Symptom Questionnaire–Short Form successfully distinguished patients with post–COVID-19 sequelae from healthy controls, achieving an accuracy of 92.18% [35].

Moreover, AI-based evaluations validated the diagnostic sensitivity of traditional questionnaires for subtle disorders such as women’s physical and mental health issues influenced by hormonal fluctuations. Researchers used support vector machine, artificial neural networks (ANNs), and decision trees to confirm the validity and accuracy of the International Physical Activity Questionnaire for menopausal women [71]. Currently, integrating multiple algorithms helps improve both model interpretability and patient classification precision. One team, for example, used a hybrid model (genetic algorithms combined with ANNs) to evaluate the effects of different weighting schemes in a traditional scale applied to daily stress events; after weight adjustments, they reported that the traditional questionnaire achieved a sensitivity of 83% and a specificity of 81% for stress detection, positioning the modified instrument as a high-performance screening tool [70].

Collectively, these findings demonstrate that AI enhances the precision with which traditional questionnaires differentiate between disease categories and unique patient populations while simultaneously refining traditional questionnaire metrics.

#### Construction of Intelligent Data Systems

**Figure 5:**
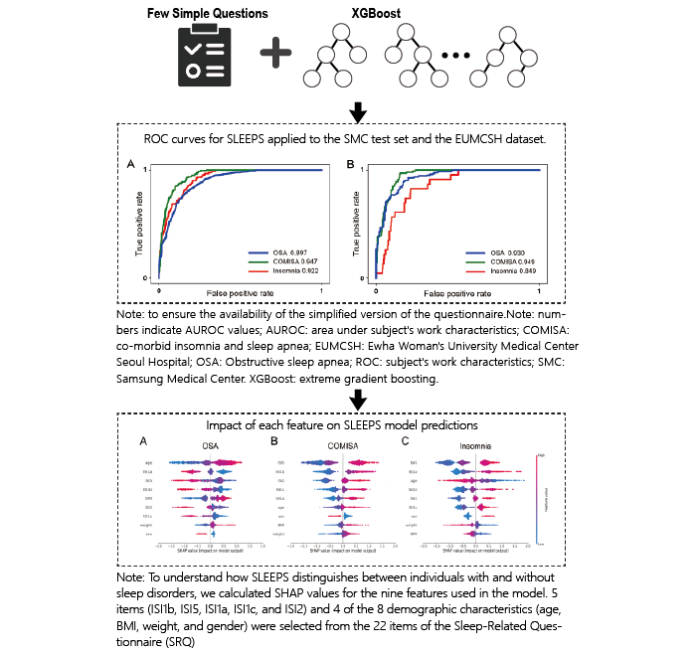
Evaluation of pathological voice data by TensorFlow and Apple’s Create ML (modified from the work by Ferreira Freitas et al [71]). AUROC: area under the receiver operating characteristic curve; COMISA: comorbid insomnia and sleep apnea; EUMCSH: Ewha Woman’s University Medical Center Seoul Hospital; ISI: Insomnia Severity Index; OSA: obstructive sleep apnea; ROC: receiver operating characteristic; SHAP: Shapley Additive Explanations; SMC: Samsung Medical Center; SRQ: Sleep Regularity Questionnaire; XGBoost: Extreme Gradient Boosting.

One of the key advantages of AI-driven data training is its capacity to construct intelligent data systems that assist clinicians in making more accurate diagnoses. Although clinical medical questionnaires undergo lengthy validation to ensure efficacy, traditional instruments often fail to comprehensively capture authentic patient behavior. For example, patients may complete questionnaires too hastily without supervision, leading to distorted results, and even after traditional assessments, diagnostic discrepancies of up to 17% may arise among different therapists [87]. Consequently, multidimensional approaches are needed in psychological and psychiatric diagnostics to reduce subjective influences and create intelligent data systems. A study on pathological voice data used AI to enhance the objectivity of the grade, roughness, breathiness, asthenia, and strain scale comparing 2 convolutional neural network (CNN)–based models—one built on Google’s TensorFlow and the other on Apple’s Core ML—both trained on identical pathological voice datasets [67]. By comparing these 2 models in classifying severity levels in pathological speech, it became possible to validate grade, roughness, breathiness, asthenia, and strain questionnaire results in real time without specialized equipment, thereby increasing the clinical objectivity of traditional questionnaires (Figure 5 [71]). In another effort, researchers designed a secure neural network–based application, randomly training and testing an ANN for diagnosing facial pain syndromes [67]. The system achieved a sensitivity of 92.4% and a specificity of 87.8%. Ultimately, AI-based data training promises to integrate patient information worldwide into intelligent online databases, thus expanding the accessibility and dissemination of intelligent data in the medical field.

#### Optimizing the Development of Medical Questionnaires Through AI

Since 2013, ANNs and genetic algorithms have been used in the development of novel medical assessment models, enhancing parameter selection for psychological questionnaires and improving the accuracy of predicting patients’ psychological states [88,89]. In 2021 and 2022, as the range of assessment methods broadened, integrating traditional ML algorithms (logistic regression [LR], RF, and decision tree) with ANN and DL tools further advanced the design of complex medical questionnaires and refined the evaluation of symptoms related to anxiety and depression (Figure 3). Nevertheless, to achieve more profound and long-term predictions of patients’ psychological conditions, it remains essential to optimize both questionnaire item formulation and the assessment processes.

#### Enhancing the Patient Applicability of Questionnaires

In developing medical questionnaires suited to diverse patient populations and conditions, AI integration confers distinct advantages. Traditional assessment methods, particularly those used to diagnose late-life depression, face notable limitations. Late-life depression is often accompanied by various comorbidities, and subtle symptom distinctions can be difficult to capture using conventional questionnaires [90-93]. To address this complexity, especially in older adults, NLP techniques can analyze patients’ linguistic patterns, thereby detecting latent symptoms that may be overlooked by traditional methods and, ultimately, expanding the questionnaire’s applicability. Moreover, Bayesian network–based analyses have been used to generate highly matched items, effectively facilitating the development of a potential risk assessment system for geriatric conditions [94,95]. AI-driven approaches have also improved applicability for other vulnerable groups. For instance, the Raghavendra Manjunath Shetty Digital Anxiety Scale leverages AI-generated facial expressions for children [68]. By enabling children to select expressions that best match their emotions, this interactive approach overcomes the limitations of traditional questionnaires, which often struggle to accurately convey or capture younger children’s emotional states.

**Figure 6:**
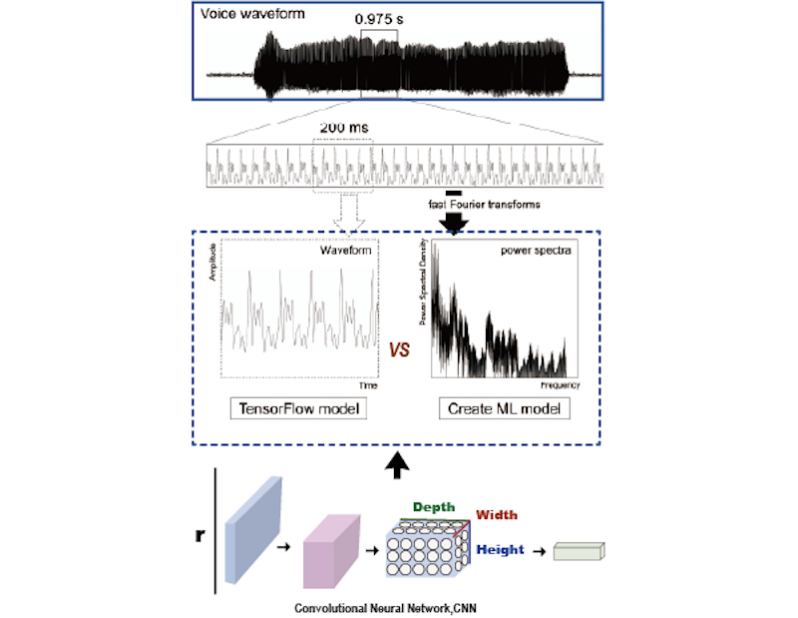
Artificial intelligence–assisted diagnostic data for system optimization and construction (modified from the work by Kojima et al [38], which is published under Creative Commons Attribution 4.0 International License [96]). CNN: convolutional neural network.

In addition, scholars have applied the extreme gradient boosting algorithm to streamline risk assessment questionnaires for insomnia and obstructive sleep apnea (OSA) [38] (Figure 6 [38,96]). By avoiding cumbersome overnight polysomnography measurements, researchers focused on feature importance to design a simplified questionnaire that accurately predicts the risks of 3 sleep disorders—OSA, comorbid insomnia and sleep apnea, and insomnia—with an area under the receiver operating characteristic curve (AUROC) exceeding 0.897 for each. Figure 6 [38] illustrates the performance of a simplified questionnaire in predicting sleep disorder risk, demonstrating high accuracy in identifying OSA (AUROC=0.897), comorbid insomnia and sleep apnea (AUROC=0.947), and insomnia (AUROC=0.922). Figure 5 [86] also highlights the influence of various features on model predictions, ultimately improving the questionnaire’s applicability across different severities of sleep disorders. By replacing complex, burdensome items with a high-confidence, streamlined set of questions, patients face a reduced response burden, and applicability to diverse patient populations is enhanced.

#### Enhancing the Cultural Adaptability of Questionnaires

In traditional clinical assessments, patients often interpret and respond differently due to varied cultural backgrounds, language habits, and life experiences. Questionnaire development must consider these cultural factors and provide timely feedback. Failure to account for the patient’s experience can result in extreme responses that skew diagnostic outcomes. Thus, incorporating appropriate open-ended questions can flexibly capture differences in patients’ understanding of medical questionnaires. One study used a multilingual and multicultural version of ChatGPT to generate a low back pain assessment questionnaire [65]. Its findings demonstrated that language training technology could effectively overcome linguistic and cultural barriers, offering strong support for future cross-cultural medical evaluations. Another study leveraged a speech dialogue system trained to develop a new pain assessment tool for patients with spinal issues [37]. By integrating natural language understanding and speech recognition, the system quickly recorded patients’ pain information and improved clinician-patient interactions. The error rates in speech recognition for physicians, nurses, and patients were 13.5%, 16.8%, and 34.7%, respectively. AI’s real-time feedback capabilities enable it to identify key factors from patient assessments across different backgrounds. One study showed that an AI-based patient-reported experience measure developed through NLP techniques could extract crucial information from patients’ open-ended responses [64], promptly relaying insights to clinicians. This timely exchange not only saves analytic time but also strengthens trust and understanding for patients from diverse cultural contexts during the diagnostic process.

#### Enhancing the Predictive Accuracy of Medical Questionnaires Through AI

Currently, AI serves as a crucial and promising tool for supporting physiological treatment and enabling early psychological interventions [97]. In predictive tasks, DL models (such as ANNs and CNNs) excel at extracting features from complex datasets, whereas ensemble learning algorithms—gradient boosting, RF, and Extreme Gradient Boosting—specialize in classification and prediction. By combining these technical strengths, AI models have transcended traditional medical constraints, effectively increasing the precision of disease prediction and diagnosis at multiple critical time points [98]. From 2022 onward, DL (CNNs and DL) and NLP (BERT, NLP, and ChatGPT) have observed growing adoption rates. Notably, the application of CNNs surged in 2023 (Figure 3), and the use of BERT and ChatGPT continued to expand in health care throughout 2024 [99]. These trends collectively underscore the advantages of integrating a range of AI methodologies to enhance predictive tasks in medical questionnaires.

#### Early Prediction of Age-Related Diseases

According to 2024 WHO data, global aging is intensifying, and chronic diseases now account for >70% of all worldwide deaths [100]. In this context, AI-enabled analysis of global patient data can facilitate disease prediction. Ophthalmological conditions such as cataracts present an increasing diagnostic burden, with surgery remaining the only effective clinical intervention. Thus, the timely identification of risk factors for patients with cataracts is essential [101-103]. A 2022 study found that DL models surpass traditional statistical models in screening accuracy for age-related diseases, enabling more rapid identification of high-risk older populations. Further research has examined the use of AI for predicting cataract surgery risk. By leveraging questionnaires and medical records, the study compared its results to those of a traditional LR model. ML models achieved an area under the curve (AUC) between 0.781 and 0.790, outperforming the LR model’s AUC of 0.767. The gradient boosting machine model demonstrated the best performance, attaining an AUC of 0.790 [101]. These findings indicate that ML can accurately forecast disease occurrence by rapidly assimilating diverse data inputs even in the absence of biological data.

#### Timely Attention to Mental Health

At present, the application of NLP techniques in mental health issue prediction is rapidly expanding [104]. ML methods (eg, LR and gradient boosting) and feature selection strategies have shown particular promise in early warning systems for mental health issues. One study on depression assessment used multiple ML and DL models to predict different levels of depressive states—normal, moderate, and severe. Among them, the RF model achieved an accuracy of 98.08%, outperforming the gradient boosting model (94.23%) and the CNN (92.31%) [103]. By applying feature selection and hyperparameter optimization, the study further enhanced model performance.

In addition, another research team used LR, RF, and gradient boosting models to evaluate suicidal intent and behaviors. Through Shapley Additive Explanations analysis, they identified the most effective features and reconstructed a simplified version of the Suicide Crisis Inventory–2 [36]. The LR model performed best, and the simplified questionnaire efficiently assessed and predicted suicidal crises in clinical settings, thereby reducing the supervision burden on health care providers [66]. The integration of AI not only improves data interpretability and predictive reliability but also contributes to timely health interventions in future health care systems.

### Potential of AI

#### Overview

The global population is projected to grow continuously by approximately 1.3 billion people between 2020 and 2050, representing a 17% increase from the 2020 population, with a peak of approximately 9.73 billion by 2064 [105]. Health informatics is a fast-growing area in the health care field. These instruments serve as critical tools for assessing population health [106]. Currently, AI technologies have already demonstrated significant potential and advantages in the development, analysis, and prediction processes of medical questionnaires.

Despite the remarkable progress of AI in this domain, numerous challenges and research gaps remain. Therefore, this section presents several new research directions based on the potential of AI to enhance rapid assessment, precise prediction, and adaptable response methods in medical questionnaires, aiming to further promote AI’s application and innovation in this field.

#### Potential for Rapid Assessment and Accurate Prediction

With the growing global population, the volume of questionnaires that health care institutions must collect and analyze is steadily increasing [107]. Traditional questionnaire evaluation methods are often time-consuming and require substantial human and economic resources [108]. Applying AI technologies to questionnaire assessments can significantly reduce labor and financial costs, a benefit that is especially pronounced in large-scale evaluations. Rapid questionnaire evaluation also enables researchers to quickly optimize questionnaires and establish related case identification systems, thereby allowing health care institutions to promptly allocate public health resources and respond more efficiently to public health events [109].

As global life expectancy continues to rise, the number and proportion of older individuals also increase. By 2030, a total of 1 in 6 people worldwide will be aged >60 years, and the population aged ≥60 years will grow from the current 1 billion to 1.4 billion, doubling to 2.1 billion by 2050 [110]. Common health issues among older adults include cognitive impairment, cataracts, and depression [111]. Integrating AI with medical questionnaires to predict disease occurrence can help family members and health care providers implement early interventions or treatments [112]. However, predictions based solely on questionnaires may have limitations. AI shows high predictive accuracy when incorporating physiological data such as speech signals, heart rate, and medical imaging [113-115]. Future research could combine questionnaire-based predictions with additional physiological information to improve and confirm the accuracy of disease forecasting.

#### New Response Modalities for Medical Questionnaires

**Figure 7:**
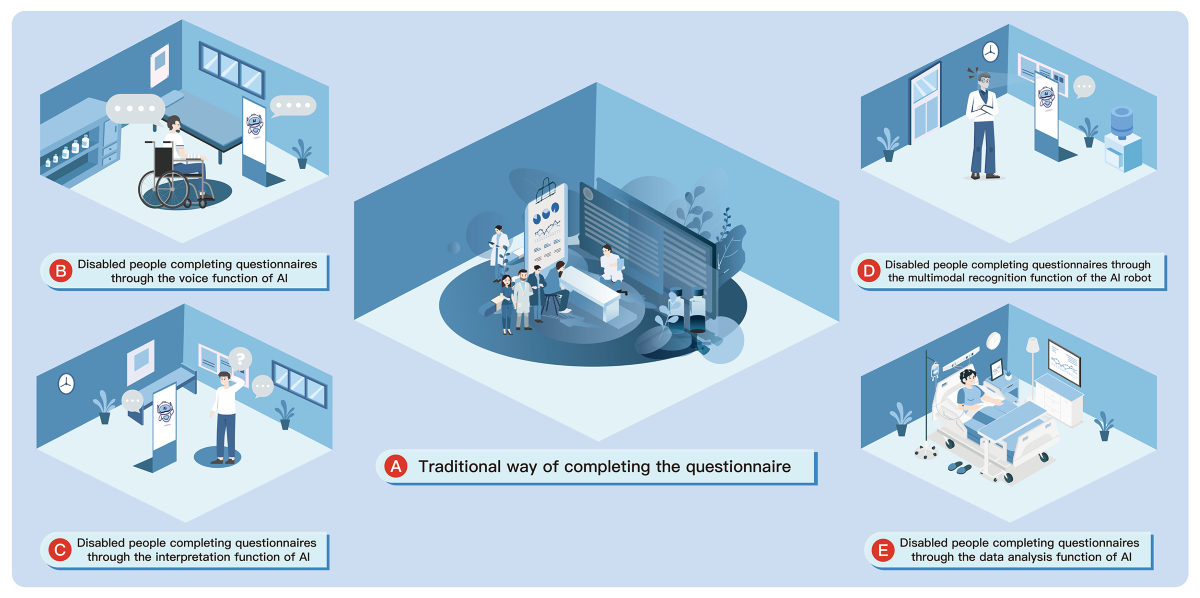
New response modalities for medical questionnaires. AI: artificial intelligence.

It is estimated that 1.3 billion people worldwide have severe disabilities, and this number is expected to rise [116]. Individuals with disabilities who have experienced stroke, limb loss, or spinal cord injuries often exhibit impaired hand function [117-119], significantly reducing their efficiency in completing traditional paper-based questionnaires. Integrating traditional questionnaires with AI-driven LLMs to create an *AI questionnaire* is one solution. By leveraging LLM technologies, users can respond verbally to questionnaire items, circumventing traditional written formats [120,121] (Figure 7).

Persons with disabilities may face discrimination in various aspects of life, resulting in generally lower educational attainment than that of other populations [122]. For these groups, especially individuals with mental disabilities such as autism or dementia, challenging vocabulary, complex sentence structures, and ambiguous answer choices pose major obstacles to questionnaire completion [123]. One approach involves using NLP-enhanced questionnaires; AI models such as ChatGPT or Bard can provide immediate explanations of terminology, thereby reducing the difficulty and improving the efficiency of questionnaire completion for individuals with lower educational levels [124,125]. Another solution integrates AI image generators such as DALL-E to add graphical support, thereby increasing the clarity and comprehensibility of questionnaire options [126]. In addition, combining virtual reality paradigms with AI-driven visual questionnaires may offer more adaptive and inclusive assessment options for individuals with cognitive limitations. This strategy could further expand the range of response modalities available in digital health evaluation [127,128].

Some individuals with mental disabilities may resist answering questionnaires through writing or speech. This reluctance can compromise the completion rate of traditional medical questionnaires, disrupt data collection, and undermine data validity. In fact, beyond textual and linguistic information, patients’ facial expressions, vocal emotions, and behaviors also carry clinically valuable data. Embodied AI robots with multimodal data collection capabilities may offer a solution [129]. By using cameras, microphones, and other devices, these robots can capture facial expressions, vocal emotional cues, and behavioral indicators during questionnaire administration [130,131]. AI-driven evaluation and analysis of these data can, in turn, guide the refinement of patient health questionnaires.

Individuals with severe disabilities—such as those with cerebral palsy, amyotrophic lateral sclerosis, or locked-in syndrome—may be unable to respond to certain medical questionnaires using normal speech or gestures for physiological or psychological reasons [132,133]. For these populations, clinicians might consider using AI technologies, brain-computer interfaces, or brainwave data to interpret and analyze patients’ eye movements, enabling more personalized completion of medical questionnaires. However, in doing so, health care professionals must ensure the accuracy of these physiological measurements [134]. Preventing the misinterpretation of patient information is essential to maintaining the validity and reliability of medical questionnaire data.

### Challenges of AI

#### Overview

AI technologies have already demonstrated substantial potential in the realm of medical questionnaires. However, the widespread implementation of AI still faces a range of complex challenges, including data privacy and security, data quality, system integration, and social equity. This section will systematically examine these issues and provide an in-depth analysis of possible strategies to overcome them. The goal is to furnish a scientific foundation and direction for effectively applying AI in medical questionnaires, thereby driving its comprehensive implementation and optimization within the health care sector.

#### Limitations of AI Technologies in Medical Questionnaires

Despite the potential that AI brings to the development, prediction, and evaluation of medical questionnaires, it also exposes multidimensional structural limitations in the context of actual clinical requirements.

ML models such as RF and LR are frequently adopted not because they represent the cutting edge of technology but because they are easier for health care professionals to understand, are less costly to validate, and do not require a reconfiguration of the hospital’s information system [135,136]. For example, Siddiqua et al [63] achieved high classification accuracy using an integrated ML model in their study of depression risk, but the choice of model was largely motivated by considerations of interpretability rather than absolute optimality of technical performance. This phenomenon suggests that the trust mechanisms required for clinical adoption are far more dominant than model complexity in many settings.

In contrast, DL models embody a different paradigm. These methods are able to mine complex patterns in high-dimensional data and even achieve learning without predefined feature structures [137]. The advantages are particularly significant when dealing with multimodal data or unstructured text [138]. However, in the specific scenario of medical questionnaires, DL models often struggle to generalize stably due to small sample sizes, high data annotation costs, and large model variance [137,139,140]. Even though ANNs performed well when used to classify facial pain in the study by McCartney et al [69], the model still faces greater challenges in clinical settings subject to legal liability due to the lack of a clear and traceable explanatory path for its diagnostic mechanism.

The emergence of natural language models, on the other hand, brings new possibilities for questionnaire development and interactive evaluation. Systems such as ChatGPT are capable of generating linguistically fluent question items and simulating preliminary physician-patient dialogues [141]. However, this class of models suffers from well-known problems such as factual errors, semantic drift, and contextual mismatch [142-144]. Coraci et al [65] validated the correlation of an AI-generated questionnaire for low back pain with a traditional scale but pointed out problems such as incomplete logic of its content and inconsistency in its scoring criteria. In the absence of strong constraints, the variability of such systems is highly likely to undermine the standardized properties on which medical questionnaires are based.

Therefore, although each of the current AI approaches has its own technical advantages, their actual value still depends on whether they can be adapted to specific clinical needs, system constraints, and resource conditions. When the task emphasizes interpretability, limited data samples, or low deployment costs, ML is still a safe choice. When the problem involves high-dimensional inputs or the identification of subtle diagnostic clues, DL is advantageous provided that the output has a verifiable path of interpretation. Natural language modeling contributes most when interactivity and content adaptation are core requirements, but it must operate under controlled mechanisms to avoid destabilizing the data. Therefore, future research should not stop at improving technical performance but should aim to build model selection strategies that respond to real clinical situations.

#### Data Privacy and Security

The application of AI in medical questionnaires necessitates handling large volumes of personal health data, making data privacy and security primary concerns [145]. The sensitivity of patient information demands stringent privacy safeguards throughout data collection, storage, and sharing processes. Federated learning has emerged as an innovative approach to protect data privacy, allowing AI models to learn from distributed datasets without centralized data sharing, thereby mitigating the risk of data breaches [146]. However, this approach still faces limitations, particularly when dealing with large-scale, heterogeneous data. In such complex scenarios, the privacy-enhancing capabilities of federated learning may be constrained [147]. Hence, future research must focus on developing more effective data privacy protection techniques. Concurrently, as AI systems become more powerful, data security requirements intensify. Related studies can further explore techniques such as homomorphic encryption and differential privacy [148], ensuring data security during transmission and processing.

In fact, safeguarding data privacy and security requires not only technical solutions but also robust legal support. Regulations such as the European Union’s General Data Protection Regulation and the US Health Insurance Portability and Accountability Act set legal standards for the legitimate use of personal health data [149]. Nonetheless, striking a balance between efficient data use and privacy protection remains a complex challenge in practice. Future research should continue exploring the equilibrium between privacy safeguards and data availability, especially when sharing data across multiple institutions. Ensuring patient information security while enhancing data usability is a key issue warranting focused attention.

#### Data Quality and Bias

As global health care systems improve, medical questionnaire data are often collected from multiple institutions. During data sharing among health care facilities, inconsistencies can arise due to variations in language translation, data formats, recording methods, and collection standards. When integrating AI into the evaluation and prediction processes for medical questionnaires, data discrepancies—such as bias, inaccuracies, or inconsistencies—can significantly undermine the reliability of AI assessments [150,151]. To ensure stable AI model performance across diverse questionnaire types, future research should consider standardizing data collection and processing methods.

Beyond data quality, data bias also critically influences the performance of AI systems in medical questionnaire development, evaluation, and prediction [152]. Bias in AI systems often stems from imbalanced training data [153]. For example, if the questionnaire data used for training underrepresent certain racial or marginalized groups or groups with certain disabilities, the model’s accuracy and predictive capabilities will diminish for these populations, potentially hindering their access to timely and effective health care services [154]. To address this issue, it is critical to ensure the diversity and representativeness of training data during questionnaire collection and development. Future research could use standardized methods such as the integration of the WHO’s Data Quality Review framework and the Metric framework designed specifically for health care AI to systematically validate the quality of training datasets [155,156]. In addition, future studies could use targeted data collection strategies such as NLP-based terminology simplification, visual questionnaire enhancement, and culturally adapted translation [157]. These might help reduce the risk of exclusion of specific populations, particularly older people, people with disabilities, and linguistically or culturally marginalized groups [158]. These combined efforts can contribute to the development of more inclusive and bias-resistant AI health assessment models.

#### Technical Limitations and Clinical Implementation Challenges

While the combination of AI with the health care clinical environment offers transformative potential for the evaluation, development, and prediction of health care questionnaires, AI continues to suffer from significant technical limitations that hinder its full application and effectiveness. The accuracy of AI diagnosis is highly dependent on the quality, quantity, and diversity of the training data [159]. If models are developed based on limited or homogeneous datasets, they tend to show a decrease in predictive performance when applied to more diverse or different patient populations, thus increasing the risk of misdiagnosis or missed diagnoses [160]. In addition, the diagnostic conclusions drawn by AI are themselves subject to a degree of uncertainty [161]. Particularly when dealing with microscopic lesions or early stages of a disease, this uncertainty may manifest itself in a high false-positive or false-negative rate, which can negatively affect clinical decision-making and may even delay patients receiving effective treatment [162].

Practical implementation challenges complicate the integration of AI-enhanced questionnaires into clinical practice, especially when considering novel response modalities (Figure 7). Integrating AI-based questionnaire tools into existing clinical workflows can be resource intensive, involving significant initial setup costs and ongoing training requirements for health care providers [163]. Clinicians are often skeptical due to the limited transparency and interpretability of AI algorithms, which further hinders the widespread adoption of AI [164,165]. Therefore, the future development of transparent and explainable AI systems and the provision of targeted training and education for health care professionals are important strategies to overcome these barriers and facilitate the successful integration of AI into clinical practice.

#### Integration With Existing Health Care Systems

For AI-based questionnaire tools to be successful in health care environments, the ability to integrate with existing health care infrastructure is critical. However, the complexity of health care systems and the lack of data interoperability pose significant challenges to such integration. Current health care systems are often intricate, with established workflows and legacy technologies that may be incompatible with new AI tools [153]. Implementing AI-based questionnaires typically requires significant infrastructure upgrades, capital investments, and time-consuming workflow redesigns. In addition, different health care institutions often use disparate data standards and formats within their electronic health record systems [166]. This variation complicates the exchange and integration of data from AI-based questionnaire tools.

Related current studies, such as the Mayo Clinic’s successful integration of an AI-based mental health screening questionnaire into its Epic electronic health record system using the Fast Healthcare Interoperability Resources standard, demonstrate advancements in health care technology [167,168]. This study significantly improved diagnostic efficiency and clinician satisfaction. Similarly, the UK National Health Service seamlessly integrated an AI-based dementia screening tool using the Health Level Seven protocol [169]. This technology streamlined clinical workflow and improved patient prognosis. These studies demonstrate that standardized interoperability frameworks can play a key role in bridging the technological divide between AI tools and clinical systems.

To facilitate scalable and sustainable AI integration, future research could prioritize standardized data formats such as the Health Level Seven or Fast Healthcare Interoperability Resources protocols to build flexible and scalable technology interfaces. Such solutions help lower the threshold of system integration and reduce the disruption to established processes, which will be beneficial in enhancing the acceptance of AI systems by medical staff and promoting the sustainable development of AI technologies in diverse health care scenarios.

#### Ethical and Regulatory Considerations

Deploying AI in health care settings raises key ethical and regulatory issues [170]. The transparency of algorithms is critical, and lack of interpretability may undermine clinician trust and potentially compromise patient safety through opaque clinical recommendations. Ensuring that AI decisions are clearly explainable and illustrative is essential to promote clinician acceptance and reduce risk [171,172].

Informed consent is also a key ethical consideration [173,174]. Patients need to be fully informed about how their data will be used by AI systems, including the potential risks and benefits, highlighting the need for transparent communication and robust consent processes. In addition, the allocation of responsibilities in AI-assisted diagnostics is an ongoing regulatory challenge [175,176]. It is essential to clearly define responsibilities among health care providers, AI developers, and organizations, particularly in the event of errors or adverse events. This requires comprehensive regulatory guidelines and liability frameworks to address the issue of liability in a transparent and effective manner.

It is important to note that the use of AI must comply with strict regulatory standards, including the General Data Protection Regulation and Health Insurance Portability and Accountability Act [177,178]. This is crucial for the legal and ethical use of AI-powered medical questionnaires. Future research should meet these stringent data protection and privacy requirements to better ensure a foundation for the safe and ethical integration of AI technologies into medical practice.

## Discussion

This study provided a comprehensive examination of the current applications, potential benefits, and challenges faced by AI in health care questionnaires. AI technologies show significant potential in all phases of questionnaire assessment, development, and prediction. In particular, AI has a positive effect on diagnostic and prognostic support in questionnaire applications for large-scale data processing, construction of personalized assessment tools, and integrated management of complex health information. These innovations highlight the transformative potential of AI in modern health care questionnaires. This is especially true in areas where medical manpower is strained, diagnostic resources are scarce, and data infrastructure is weak. Lightweight ML models and automated questionnaire processes can take on initial screening and triage functions, alleviating manpower pressure and improving early identification. AI questionnaire systems with adaptive capabilities can improve the relevance of questions and reduce the burden of answering in environments where professionals are lacking.

Although AI has demonstrated great advantages in its application to medical questionnaires, its interpretability and adoption mechanisms in clinical decision-making still lack systematic understanding. Questionnaire designs with dynamic logic capabilities still face challenges in maintaining measurement validity, especially in user interaction scenarios that emphasize semantic consistency. Developing AI models that are maintainable and adaptable to deployment in resource-constrained environments also remains a key challenge. In addition, data privacy, data quality bias, and ethical and regulatory barriers are still the core issues constraining the implementation of the technology.

This review has several limitations. First, the number of eligible studies was small, which may restrict the generalizability of the findings. Second, most included studies were of moderate methodological quality, with limited clinical validation and frequent lack of control groups or standardized evaluation metrics. Third, the review was restricted to English- and Chinese-language literature, potentially omitting relevant studies in other languages. Lastly, the heterogeneity of AI models, clinical contexts, and questionnaire types limited the ability to perform quantitative synthesis or direct comparison across studies.

In response to the aforementioned challenges, future research should further promote the evolution of AI systems toward interpretability, contextual adaptability, and cross-platform compatibility and promote the establishment of a more standardized, safe, and controllable AI architecture. The validation of the applicability of AI-based questionnaire tools under diverse health systems should be strengthened, and localized deployment strategies for low-resource environments should be developed. Through the focus on these key issues, AI is expected to continue to drive the overall improvement of health care questionnaires in terms of diagnostic efficiency, patient engagement, and health equity.

## Appendix

Multimedia Appendix 1 PRISMA-ScR Checklist.

Multimedia Appendix 2 Advances in smart technology in the clinical and research environments in the included studies (N=14).

## Abbreviations

AI: artificial intelligence
ANN: artificial neural network
AUC: area under the curve
AUROC: area under the receiver operating characteristic curve
BERT: bidirectional encoder representations from transformers
CNN: convolutional neural network
DL: deep learning
JBI: Joanna Briggs Institute
LLM: large language model
LR: logistic regression
ML: machine learning
NLP: natural language processing
OSA: obstructive sleep apnea
PRISMA-ScR: Preferred Reporting Items for Systematic Reviews and Meta-Analyses extension for Scoping Reviews
RF: random forest
WHO: World Health Organization

## Data Availability

Data sharing is not applicable to this article as no datasets were generated or analyzed during this study.
