# Correction: AI in Medical Questionnaires: Innovations, Diagnosis, and Implications

**DOI:** 10.2196/80644

**Published:** 2025-08-11

In the article, “AI in Medical Questionnaires: Innovations, Diagnosis, and Implications,” [1] published June 23, 2025, errors in the article type and manuscript content were not corrected during editing of the article. The JMIR Publications Editorial Office issues this corrigendum due to the resulting inconsistencies in the manuscript with the appropriate article type format and content.

Post-publication corrections have been made to correct the manuscript:

- The article type is changed from Viewpoint to Review.
- The article title is revised from “AI in Medical Questionnaires: Innovations, Diagnosis, and Implications” to “AI in Medical Questionnaires: Scoping Review.”
- The abstract is corrected to reflect the scoping literature review article type.
- The manuscript text is corrected and reorganized to reflect the scoping literature review article type, and some references are renumbered accordingly.
- In the Methods, reference 61 is corrected to reference PRISMA-ScR guidance.
- Table 2, Figure 1, and their associated text are moved from the Methods to the Results, and renumbered accordingly.
- Current Figure 1 caption is added, and the figure is renumbered.
- Current Figure 2 is corrected, and the figure is renumbered.
- Current Figure 3 is corrected, and the figure is renumbered.
- Authors added discussion of limitations to the manuscript.
- A new multimedia appendix is added, which is an author-completed PRISMA-ScR checklist.

The correction will appear in the online version of the paper on the JMIR Publications website, together with the publication of this correction notice. Because this was made after submission to PubMed, PubMed Central, and other full-text repositories, the corrected article has also been resubmitted to those repositories.
