# Supplementary material for: AI in Medical Questionnaires: Scoping Review
Source: J Med Internet Res. 2025 Jun 23;27:e72398. doi: 10.2196/72398 (PMC12235208; doi:10.2196/72398)
Supplement: Multimedia Appendix 2 [file jmir_v27i1e72398_app2.docx]

**Multimedia appendix 1** Advances in Smart Technology in Clinical and Research in Key Texts(N=14)

| **ID** | **Aims** | **Measurements** | **Functions** | **Outcomes** | **Clinical/Research** |
| --- | --- | --- | --- | --- | --- |
| 1 | Depression assessment | BDI,HDRS,MADRS,EQ-5D,PHQ-9, QIDS-SR,DASS-21, K-10. | Voting algorithm, Machine Learning (Random Forest, Gradient Boosting, CNN), Hyperparameter optimizers, Feature selection methods, LIME. | High prevalence of depression among youths, early diagnosis helps in providing effective treatment, identification of factors contributing to depression | Research |
| 2 | Patient experience assessment | AI-PREM (open-ended questionnaire) | NLP pipeline (sentiment analysis and topic modeling) | High representativeness of topics and a 90% overlap between automatically and manually extracted topics | Clinical |
| 3 | Development of medical questionnaires for low back pain | ChatQ, ODI, QBPDS, RMDQ, NRS. | AI language model, content comparison with validated questionnaires | Chatqpt showed significant correlation with ODI and QBPDS, acceptable for assessing low back pain | Research |
| 4 | Development of a SDS for spinal pain assessment | Spinal pain questionnaire protocol | NLU, Speech Recognition, Dialogue Management, NLG, Speech Synthesis | Favorable user satisfaction and performance accuracy in medical staff and patients | Clinical |
| 5 | Development of a predictive tool for suicide risk | SCI-2 | Machine learning algorithms (Logistic Regression, Random Forest, Gradient Boosting); SHAP analysis | The SCI-2 Short Form was developed with good predictive performance | Research |
| 6 | Distinguish Long COVID, ME/CFS, and healthy controls | DSQ-SF | Random forest machine learning algorithms | The DSQ-SF can accurately classify these populations with high sensitivity and specificity. | Research |
| 7 | Objective assessment of pathological voice | GRBAS scale | Machine Learning models using TensorFlow and Core ML | Both models performed well in G and B, and AI was considered a sufficiently reliable method for medical screening purposes | Research |
| 8 | Predicting the 10- year risk of cataract surgery | Self-reported questionnaire data | ML algorithms (RF, GBM, DL), Logistic model, AUCs. | ML models outperformed traditional logistic regression for cataract surgery prediction | Clinical |
| 9 | Predicting the risk of sleep disorders (OSA, COMISA, Insomnia) | Self-reported questionnaire data (ISI, ESS, PSQI) | XGBoost, SHAP for feature importance | High accuracy in predicting sleep disorders with AUC > 0.897 for all disorders | Research |
| 10 | Assessing dental anxiety in children | RMS-DAS, RMS-PS, FIS | AI-based expressions, image processing | RMS-DAS is reliable and valid for assessing dental anxiety in children | Research |
| 11 | Diagnosing facial pain syndromes | Facial pain questionnaire responses | Artificial neural network (ANN) | ANN correctly diagnoses TN1 with high sensitivity and specificity | Research |
| 12 | Validation of the Revised Stressful Life Event Questionnaire | Stressful Life Event Questionnaire, GHQ-12 | GA, ANNs | Death of parents, spouse, or siblings is the most impactful stressor | Research |
| 13 | Validity and precision of the IPAQ for climacteric women | IPAQ | Kohonen and k-means algorithms, Silhouette, PBM, and Dunn indexes | IPAQ categorizes results into sufficiently active and insufficiently active. | Research |
| 14 | CGA | CGA Tool | Bayesian Network, Questionnaire Algorithm | Superior performance in accuracy, comprehensive performance, and space occupancy compared to FPQM | Research |

Abbreviation: **BDI** = Beck Depression Inventory; **HDRS** = Hamilton Depression Rating Scale; **MADRS** = Montgomery-Åsberg Depression Rating Scale; **EQ-5D** = EuroQol-5 Dimensions Questionnaire; **PHQ-9** = Patient Health Questionnaire-9; **QIDS-SR** = Quick Inventory of Depressive Symptomatology – Self-Report; **DASS-21** = Depression, Anxiety and Stress Scales; **K-10** = Kessler Psychological Distress Scale; **CNN** = Convolutional Neural Network; **LIME** = Local Interpretable Model-Agnostic Explanations; **AI-PREM** = Artificial Intelligence Patient-Reported Experience Measure; **NLP** = Natural Language Processing; **F1 score** = A measure of a model's accuracy, specifically in binary classification tasks; **SCI-2** = Suicide Crisis Inventory-2; **SHAP** = SHapley Additive Explanation; **DSQ-SF** = DePaul Symptom Questionnaire-Short Form; **ME/CFS** = Myalgic Encephalomyelitis/Chronic Fatigue Syndrome; **MDA** = Mean Decrease in Predictive Accuracy (a measure used in random forest algorithms); **OSA** = Obstructive Sleep Apnea; **COMISA** = Comorbid Insomnia and Sleep Apnea; **ISI** = Insomnia Severity Index; **ESS** = Epworth Sleepiness Scale; **PSQI** = Pittsburgh Sleep Quality Index; **XGBoost** = Extreme Gradient Boosting; **AUC** = Area Under the Receiver Operating Characteristic Curve; **RMS-DAS** = Raghavendra Manjunath Shetty’s Digital Anxiety Scale; **RMS-PS** = Raghavendra Manjunath Shetty’s Pictorial Scale; **FIS** = Facial Image Scale; **Cronbach’s α**= Cronbach's alpha, a measure of internal consistency reliability; **PROMs** = Patient-Reported Outcome Measures; **PREMs** = Patient-Reported Experience Measures; **VASs** = Visual Analogue Scales; **GHQ-12** = General Health Questionnaire-12; **GA** = Genetic Algorithm; **ANNs** = Artificial Neural Networks; **CGA** = Comprehensive Geriatric Assessment; **FPQM**: Fast Preceding Questionnaire Model. **ChatQ** = ChatGPT questionnaire; **ODI** = Oswestry Disability Index; **QBPDS** = Quebec Back Pain Disability Scale; **RMDQ** = Roland-Morris Disability Questionnaire; **NRS** = Numerical Rating Scale; **SDS** = Spoken Dialogue System; **NLU** = Natural Language Understanding; **NLG** = Natural Language Generation; **ME/CFS** = Myalgic Encephalomyelitis/Chronic Fatigue Syndrome; **MDA** = Mean Decrease in Predictive Accuracy (a measure used in random forest algorithms); **GRBAS** = Grade, Roughness, Breathiness, Asthenia, and Strain scale; **ML** = Machine Learning; **TensorFlow** = An open-source machine learning framework developed by Google; **Core ML** = A machine learning framework developed by Apple; **RF** = Random Forests; **GBM** = Gradient Boosting Machine; **DL** = Deep Learning; **AUCs** = Areas Under the Receiver Operating Characteristic Curve, a measure used to evaluate the performance of a binary classifier system; **TN1** = Trigeminal Neuralgia Type 1; **ANN** = Artificial Neural Network; **IPAQ** = International Physical Activity Qu ; **PBM** = The maximum value of this index
